# Inhibition of BRD4 activates the AKT-SIRT3 signaling pathway to suppress apoptosis and attenuate hyperoxia-induced lung injury

**DOI:** 10.3389/fbioe.2025.1674916

**Published:** 2025-11-07

**Authors:** Kangjie Qin, Jie Zheng, Yuting Zhang, Yiyu Wang, Han Qin, Qiuyu Dai, Xinxin Liu, Liting Cheng, Kun Yu, Miao Chen, Song Qin

**Affiliations:** 1 Department of Critical Care Medicine, Affiliated Hospital of Zunyi Medical University, Zunyi, China; 2 Department of Respiratory and Critical Care Medicine, Kweichow Moutai Hospital, Renhuai, Guizhou, China; 3 Zunyi Medical University, Zunyi, China; 4 Department of Critical Care Medicine, Kweichow Moutai Hospital, Renhuai, Guizhou, China

**Keywords:** hyperoxia-induced lung injury, apoptosis, Akt, sirt3, AEC-II cells

## Abstract

As a critical pulmonary complication in oxygen therapy, hyperoxia-induced lung injury (HILI) is featured with edema, alveolar wall thickening, and inflammatory cell infiltration. Bromodomain containing 4 (BRD4) has been documented as a vital regulator of apoptosis, inflammation, and oxidative stress under various pathological conditions. However, whether BRD4 plays a part in HILI has not yet been well investigated. The current investigation revealed a significant elevation of BRD4 expression in both *in vitro* and *in vivo* models of HILI. Notably, BRD4 knockdown effectively attenuated apoptosis, oxidative stress, and inflammatory response in H_2_O_2_-challenged AEC-II cells. Further investigation elucidated that BRD4 knockdown activated the AKT signaling pathway and upregulated SIRT3 expression *in vitro* and *in vivo*. AKT inhibition markedly abrogated BRD4 silencing-mediated AKT activation and SIRT3 upregulation in AEC-II cells exposed to H_2_O_2_, while SIRT3 inhibition failed to alter AKT activation. In addition, AKT inactivation also reversed BRD4 inhibition-mediated increased in the transcriptional activity of SIRT3. Furthermore, AKT inactivation or SIRT3 inhibition significantly diminished the protective effects of BRD4 knockdown on H_2_O_2_-treated AEC-II cells. In summary, this work elucidated that BRD4 inhibition ameliorates HILI through AKT-mediated SIRT3 upregulation. Our study highlights the vital role of the BRD4/AKT/SIRT3 axis in mediating HILI and suggests BRD4 as an attractive target for HILI management.

## Introduction

Oxygen supplementation is widely applied to alleviate severe respiratory failure in critical pneumonia and acute lung injury (ALI) patients (Sohn et al., 2010). However, sustained high-concentration oxygen exposure paradoxically induces systemic oxidative damage, with the pulmonary system being particularly susceptible to oxygen toxicity (Hong et al., 2021; Huang et al., 2024). Hyperoxia-induced ALI (HILI) is marked by edema, alveolar wall thickening, and inflammatory cell infiltration (Sun et al., 2025). Recent studies suggest that inhibition of ferroptosis alleviates hyperoxia-induced lung injury (Guo et al., 2022), while induction of ferroptosis may serve as a therapeutic approach for tumors (Ma et al., 2024; Guo K. et al., 2023). Despite advancements in supportive care, effective pharmacological interventions targeting the underlying molecular mechanisms of HILI remain elusive.

Covering the alveolar surface are predominantly alveolar type II epithelial cells (AEC-II cells), which maintaining the homeostasis within the lungs through the synthesis, secretion and circulation of pulmonary surfactant (Hernández-Hernández et al., 2024). During HILI, hyperoxia induces oxidative stress and mitochondrial dysfunction, resulting in apoptosis and inflammation of AEC-II cells and subsequent lung tissue damage (Liu G. et al., 2019). AEC-II cells are the primary target of hyperoxia-induced lung injury (Lee and Kim, 2011). Hyperoxia induces reactive oxygen species (ROS) production and promotes apoptosis in AEC-II cells (Yang et al., 2022). Therefore, preserving the viability and functionality of AEC-II cells represents a promising approach for HILI treatment.

In recent years, increasing efforts have been devoted to uncovering the molecular pathways involved in HILI, with the goal of discovering novel therapeutic targets for more effective interventions. Among the potential candidates, bromodomain-containing protein 4 (BRD4), a member of the bromodomain and extra-terminal domain (BET) family, has emerged as a particularly promising target (Jin et al., 2018; Li et al., 2024). BRD4 possesses the ability to selectively bind acetylated lysine residues on both histone and non-histone proteins through its bromodomains, thereby recruiting transcriptional regulatory complexes to regions of acetylated chromatin (Shi and Vakoc, 2014; Wu et al., 2024). Functioning as both an epigenetic modulator and a transcriptional coactivator, BRD4 plays a pivotal role in controlling key cellular processes such as apoptosis, inflammation, and oxidative stress across various pathological conditions (Zhang and Xu, 2020; Huang et al., 2025). For example, inhibition or degradation of BET bromodomain proteins such as BRD4 results in DNA damage and apoptosis in cells (Lam et al., 2020). Previous studies suggest that Inhibition of BRD4 alleviates acute kidney injury-oxidative stress and apoptosis by PI3K/AKT pathway (Liu H. et al., 2019), SIRT3 via regulating autophagy and apoptosis through the PI3K/Akt pathway (Xu et al., 2021). Emerging evidence highlights BRD4 as a potential therapeutic target in pulmonary diseases (Guo X. et al., 2023; Li et al., 2025). However, the studies of the BRD4/AKT/SIRT3 signaling pathway in pulmonary diseases remains relatively limited. Whether BRD4 is involved in HILI pathogenesis remains elusive.

In this study, we present compelling evidence demonstrating the protective efficacy of BRD4 inhibition against HILI, with a focus on the AKT-SIRT3 signaling pathway.

By integrating molecular and histopathological approaches, this study aimed to clarify the functional role of BRD4 in alleviating apoptosis, inflammation, and oxidative stress associated with HILI, both *in vitro* and *in vivo*. We hypothesized that BRD4 inhibition would attenuate HILI by repressing these pathological processes through activation of the AKT-SIRT3 signaling pathway. Exploring the complex interplay between BRD4, AKT, and SIRT3 in HILI could provide important perspectives on innovative treatment approaches for HILI control.

## Materials and methods

### Isolation of AEC-II cells

As previously mentioned (Ballinger et al., 2012), AEC-II cells were extracted and grown in DMEM/F12 (10% FBS) under suitable conditions (37 °C; 5% CO_2_).

Since H_2_O_2_ can induce oxidative stress and cellular responses as seen in HILI, H_2_O_2_-induced AEC-II cells are often used as a cellular model to simulate HILI *in vitro* (Qin et al., 2019; Sturrock et al., 2012). Briefly, AEC-II cells were challenged by H_2_O_2_ (500 μM) for 24 h (Wang et al., 2008). For AKT inactivation, AEC-II cells were treated with AKT inhibitor, Ly294002 (10 μM; Selleck Chemicals, United States of America), 2 h before H_2_O_2_ treatment. For SIRT3 inhibition, AEC-II cells were treated with SIRT3 inhibitor, 3-TYP (5 μM; Selleck Chemicals), 2 h prior to H_2_O_2_ treatment.

#### CCK-8

AEC-II cell viability was analyzed via CCK-8 assay. Cells were first placed in 96-well plates (8 × 10^3^ cells/well). Following certain treatments, CCK-8 reagent (Dojindo, Japan) was applied to each well (10 μL/well) and further incubated for 2 h. The absorbance at 450 nm was measured using a microplate reader.

### Flow cytometry

The apoptosis of AEC-II cells was assessed using a FITC-labeled Annexin V Kit (BD Pharmingen) by flow cytometry. The treated AEC-II cells were harvested, washed, centrifuged, and suspended in PBS. Next, the cells were incubated with FITC-labeled Annexin V and PI for 30 min at 37 °C before being analyzed using a FACSCalibur flow cytometer.

### Cell transfection

BRD4 overexpression vector (BRD4-OE), empty vector (Vector), short hairpin RNA (shRNA) targeting BRD4 (sh-BRD4), SIRT3 (sh-SIRT3), and negative control (sh-NC) were synthesized by GenePharma (Shanghai, China). Briefly, AEC-II cells were transfected with the plasmids mentioned above using Lipofectamine™ 3,000 and cultured for 48 h before further experiments.

### RT-qPCR

Via Trizol reagent (Invitrogen), total RNA was extracted from lung tissues or AEC-II cells and reversely transcribed into cDNA using PrimeScript RT reagent Kit (TaKaRa). The cDNA amplification and recording were conducted using HieffTM qPCR SYBR® Green Master Mix in an ABI 7500 Real-Time PCR System (Applied Biosystems). Relative gene expression was calculated by the 2^−ΔΔCT^ method after normalization to GAPDH.

### Western blotting

Total proteins were isolated from AEC-II cells or lung tissue samples using an appropriate lysis buffer. Equal amounts of protein were subjected to 10% SDS-PAGE for electrophoretic separation, followed by transfer onto PVDF membranes. The membranes were then blocked with 5% skim milk at room temperature for 1 h, incubated with primary antibodies at 4 °C overnight, and subsequently exposed to HRP-conjugated secondary antibodies for 2 h at room temperature. Blot bands were developed by ECL reagents (Amersham, United Kingdom) and quantified with ImageJ software.

### LDH assay

AEC-II cell damage was determined by measuring LDH release utilizing the LDH Release Assay Kit (Beyotime).

## ELISA

The concentrations of TNF-α and IL-6 in bronchoalveolar lavage fluid (BALF) and cell culture supernatant were detected by utilizing ELISA kits.

### Measurements of malondialdehyde (MDA), superoxide dismutase (SOD) and glutathione (GSH) levels

The MDA, SOD, and GSH levels in cells and tissue samples were detected using corresponding commercial kits (Jiancheng, China) according to the standard protocol.

### Determination of SIRT3 deacetylase activity

As described previously (Wang et al., 2020), the deacetylase activity of SIRT3 was evaluated by utilizing the SIRT3 Activity Assay Kit (Abcam). The fluorescence intensity was determined with a microplate reader.

### Dual-luciferase reporter assay

A SIRT3 promoter reporter plasmid was synthesized by Sangon Biotech (Shanghai, China). This construct was transfected into AEC-II cells, either alone or in combination with sh-BRD4. 48 h later, transfected AEC-II cells were subject to indicated treatment. Luciferase activity was measured using the Dual-Luciferase Reporter Assay System (Promega) according to the manufacturer’s protocol.

### Establishment of the HILI model

To establish the HILI model, C57BL/6 mice of either sex (6–8 weeks old) were used in the study, which obtained from the Model Animal Research Center of Nanjing University. And the experiments were conducted in a blinded manner. Animals were maintained under standard laboratory conditions with unrestricted access to food and water. The mice were randomly divided into Control, HILI, and HILI + JQ1 groups. As previously described (Qin et al., 2018). HILI mice were exposed to oxygen at a high concentration of (≥95%; 5.0 L/min) for 72 h. For BRD4 inhibition *in vivo*, a BET bromodomain inhibitor JQ1 (Selleck Chemicals) was administrated to HILI mice. Mice received intraperitoneal injection of JQ1 (dissolved in DMSO solution; 25 mg/kg; ApexBio, United States of America) 1 h before HILI modeling. Then, the mice were humanely euthanized via intraperitoneal administration of an overdose of pentobarbital sodium (150 mg/kg), after which bronchoalveolar lavage fluid (BALF) and lung tissues were collected for further analysis. All animal procedures were permitted by the Affiliated Hospital of Zunyi Medical University (KLL-2021-095).

### Hematoxylin and eosin (H&E) staining

Briefly, lung tissues were immersed in 4% paraformaldehyde, embedded in paraffin, and then sliced into 5 μm slices. Next, slices were deparaffinized, rehydrated, and stained with hematoxylin and eosin. Histological examination was performed using a light microscope (Olympus, Japan).

### Lung wet/dry (W/D) weight ratio

The right lung was excised from each mice, gently blotted to remove surface moisture, and immediately weighed to obtain the wet weight. The samples were then dried in an oven at 80 °C until a constant weight was achieved, representing the dry weight. The W/D ratio was calculated as an indicator of pulmonary edema.

### TUNEL assay

The apoptosis in lung tissues was detected using a TUNEL assay kit (Roche, United States of America). Apoptotic cells were visualized under a fluorescence microscope, and quantification was performed by randomly selecting five microscopic fields per section and counting TUNEL-positive cells.

### Statistical analysis

Graphs were plotted with GraphPad 9.0 software. All data were obtained from at least three independent experiments and were expressed as mean ± standard deviation. One-way analysis of variance (ANOVA) with Tukey multiple comparison test is used for data analysis. Any difference with a P value <0.05 was considered statistically significant.

## Results

### BRD4 is upregulated in H_2_O_2_-treated AEC-II cells

**FIGURE 1:**
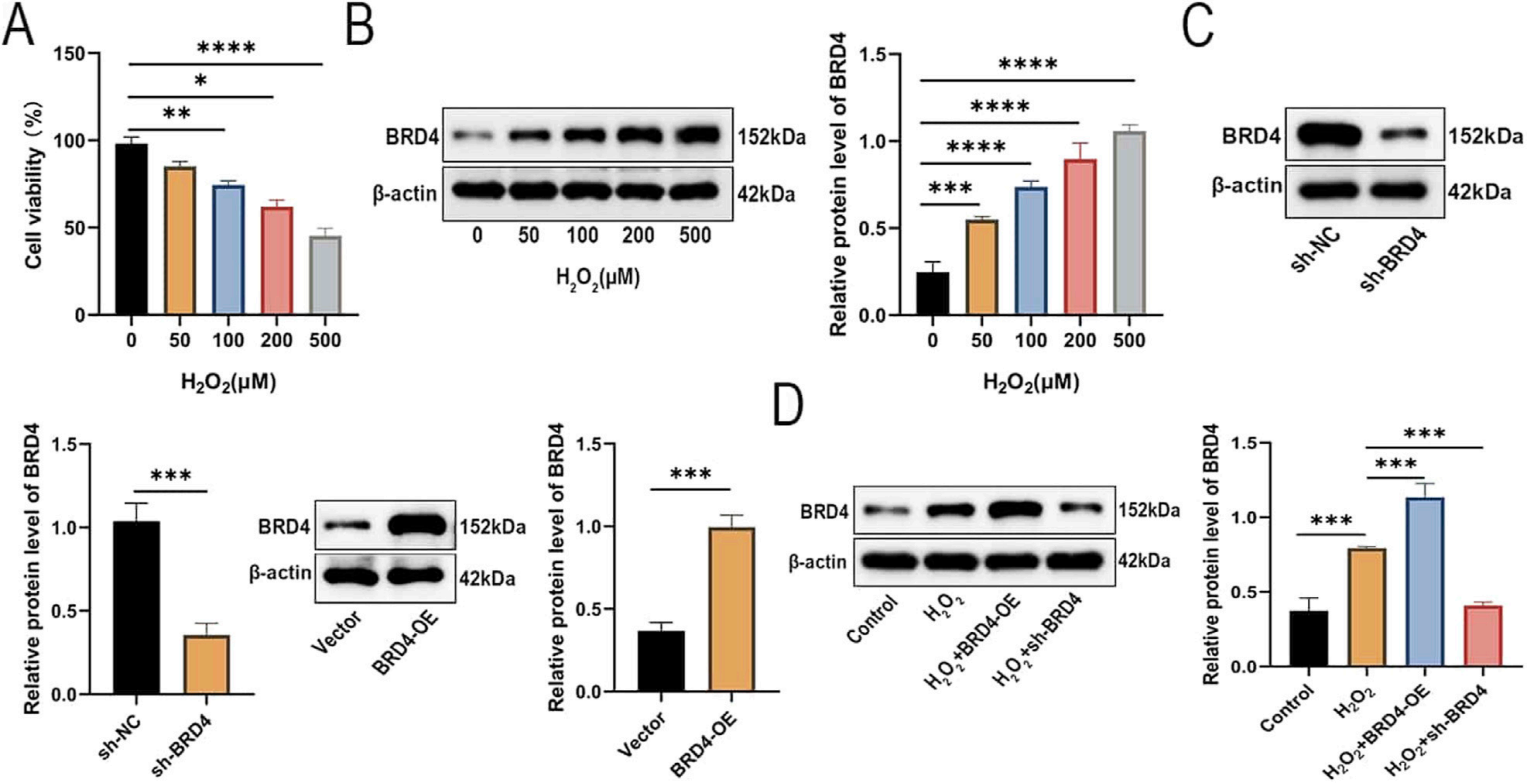
BRD4 is upregulated in H_2_O_2_-treated AEC-II cells. AEC-II cells were exposed to increasing concentrations of H_2_O_2_ (0, 50, 100, 200, 500 μM) and cultured for 24 h. **(A)** Cell viability of AEC-II cells was detected by CCK-8 assay. **(B)** The BRD4 protein level in **AEC**-II cells exposed to different concentrations of H_2_O_2_ was detected by Western blotting. **(C)** The BRD4 protein level in AEC-II cells transfected with sh-NC, sh-BRD4, Vector, or BRD4-OE. **(D)** Transfected AEC-II cells were exposed to 500 μM H_2_O_2_ and cultured for 24 h. BRD4 protein level in AEC-II cells from Control, H_2_O_2_, H_2_O_2_+BRD4-OE, and H_2_O_2_+sh-BRD4 groups. Cell experiments were performed in triplicates (n = 3). *P < 0.05; **P < 0.01; ***P < 0.001; ****P < 0.0001.

First, we explored whether treatment with H_2_O_2_ resulted in a significant change in AEC-II cell viability and was associated with altered expression of BRD4. Relative to the control group, H_2_O_2_ exposure dose-dependently reduced the survival of AEC-II cells (Figure 1A). As shown in Figure 1B, H_2_O_2_ exposure significantly promoted BRD4 expression. Then, Western blotting results confirmed the successful knockdown or overexpression of BRD4 in AEC-II cells (Figure 1C). AEC-II cells were challenged by H_2_O_2_ (500 μM) for 24h, As expected, BRD4 overexpression augmented H_2_O_2_-induced BRD4 upregulation in AEC-II cells, while BRD4 knockdown significantly inhibited BRD4 expression (Figure 1D). These results confirmed that BRD4 was significantly increased in H_2_O_2_-treated AEC-II cells.

### BRD4 regulates H_2_O_2_-induced inflammatory responses, oxidative stress, and apoptosis in AEC-II cells

**FIGURE 2:**
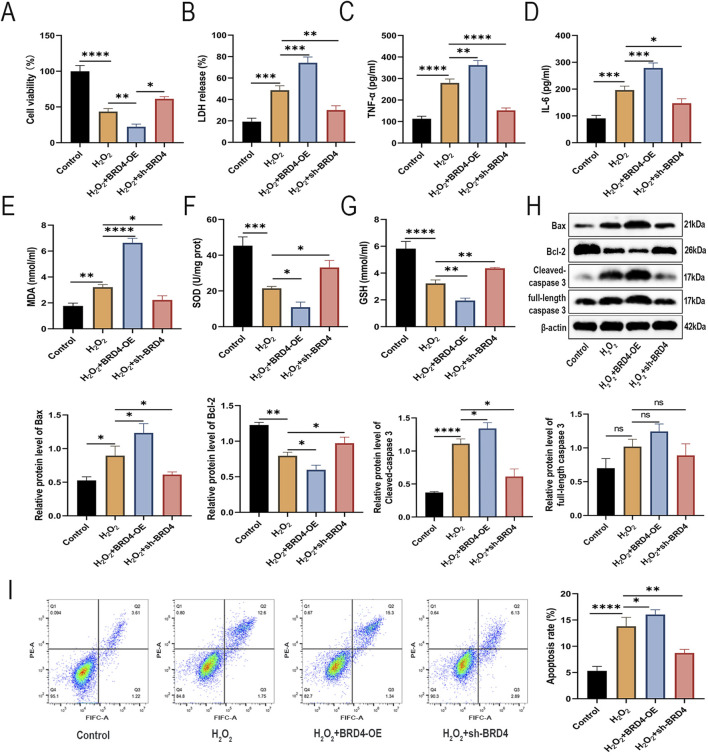
BRD4 regulates H_2_O_2_-induced inflammatory responses, oxidative stress, and apoptosis in AEC-II cells. AEC-II cells were assigned to Control, H_2_O_2_, H_2_O_2_+BRD4-OE, and H_2_O_2_+sh-BRD4 groups. **(A,B)** The viability and LDH release of AEC-II cells from each group. **(C,D)** TNF-α and IL-6 levels in AEC-II cell supernatant from each group were detected by ELISA. **(E–G)** MDA, SOD, and GSH levels in AEC-II cells from each group. **(H)** Bax, Bcl-2, full-length caspase 3, and cleaved-caspase 3 protein levels in AEC-II cells from each group. **(I)** The apoptosis of AEC-II cells from each group was detected by flow cytometry. Cell experiments were performed in triplicates (n = 3). *P < 0.05; **P < 0.01; ***P < 0.001; ****P < 0.0001.

Next, we investigated the effects of BRD4 on H_2_O_2_-induced **AEC**-II cells. As illustrated in Figures 2A,B,H_2_O_2_ treatment significantly reduced **AEC**-II cell proliferation and caused cell damage, which was significantly reversed by BRD4 knockdown but further enhanced by BRD4 overexpression. ELISA analysis showed that BRD4 knockdown suppressed, while its overexpression promoted, H_2_O_2_-induced TNF-α and IL-6 release in AEC-II cells (Figures 2C,D). Furthermore, H_2_O_2_-induced malondialdehyde (MDA) was decreased by BRD4 knockdown but increased by BRD4 overexpression; meanwhile, H_2_O_2_-triggered decrease in superoxide dismutase (SOD) and glutathione (GSH) was abolished by BRD4 inhibition but potentiated by BRD4 overexpression (Figures 2E–G). In addition, H_2_O_2_ treatment increased Bax, both full-length caspase 3 and cleaved-caspase 3 protein levels in **AEC**-II cells; notably, these effects were effectively abrogated by BRD4 suppression but reinforced by BRD4 overexpression (Figure 2H). Similarly, BRD4 depletion inhibited H_2_O_2_-triggered apoptosis in **AEC**-II cells; however, BRD4 further aggravated H_2_O_2_-induced **AEC**-II cell apoptosis (Figure 2I). Therefore, BRD4 knockdown inhibits H_2_O_2_-triggered apoptosis of **AEC**-II cells.

### BRD4 inhibition attenuates H_2_O_2_-Induced AEC-II cell injury via AKT pathway activation

**FIGURE 3:**
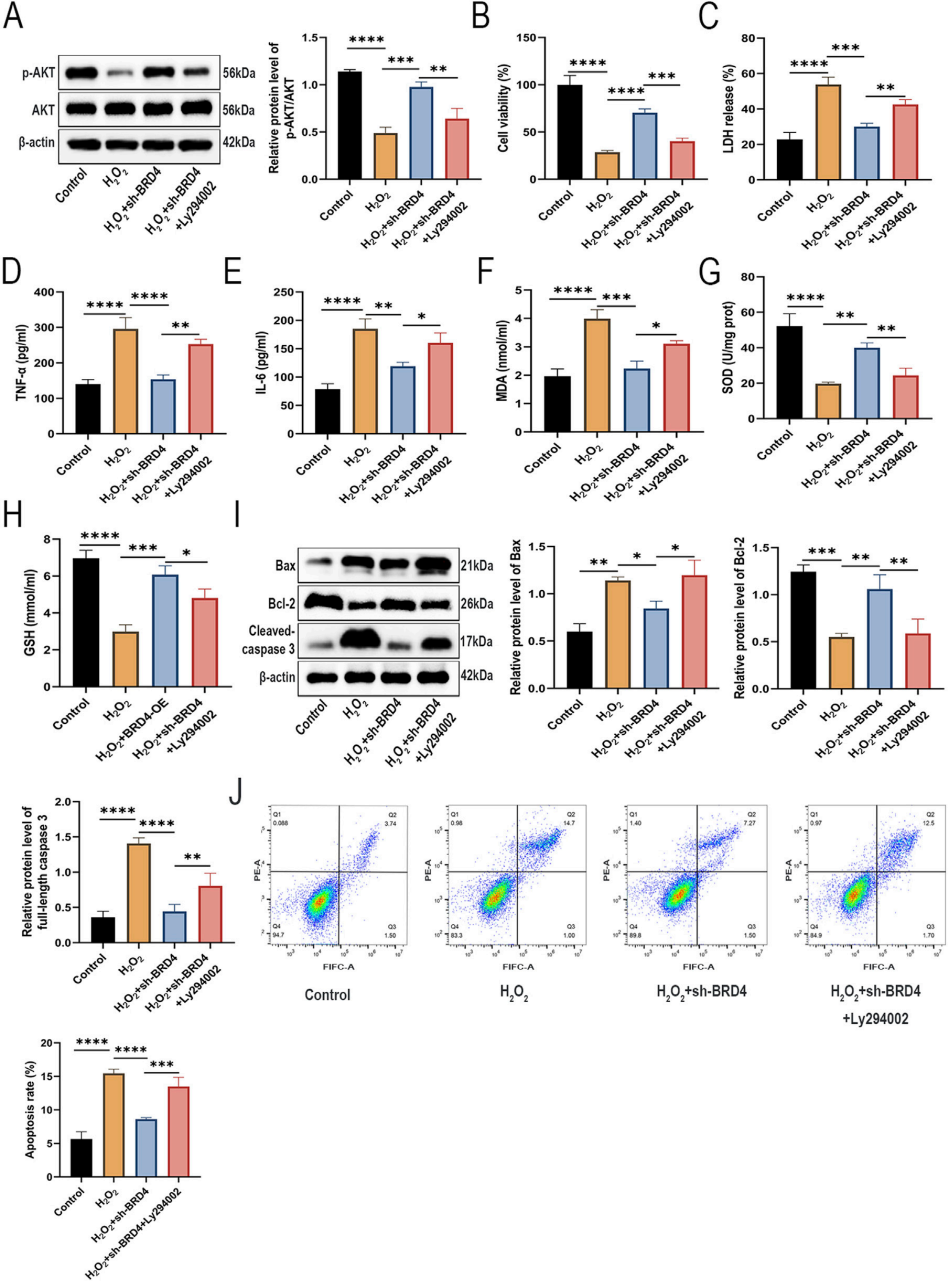
Knockdown of BRD4 alleviated H_2_O_2_-induced injury in AEC-II cells through activation of the AKT pathway. AEC-II cells were assigned to Control, H_2_O_2_, H_2_O_2_+sh-BRD4, and H_2_O_2_+sh-BRD4+Ly294002 groups. **(A)** The p-AKT and AKT protein levels in AEC-II cells from each group. **(B,C)** The viability and LDH release of AEC-II cells from each group. **(D,E)** TNF-α and IL-6 levels in AEC-II cell supernatant from each group. **(F–H)** MDA, SOD, and GSH levels in AEC-II cells from each group. **(I)** Bax, Bcl-2, and cleaved-caspase 3 protein levels in AEC-II cells from each group. **(J)** The apoptosis of AEC-II cells from each group. Cell experiments were performed in triplicates (n = 3). *P < 0.05; **P < 0.01; ***P < 0.001; ****P < 0.0001.

Activation of the AKT pathway plays a pivotal role in promoting cell growth and preventing apoptosis (Qiu et al., 2023). In addition, AKT pathway activation has been reported to protect against AEC-II cell apoptosis in HILI (Soutar et al., 2018). Interestingly, BRD4 inhibition can alleviate oxidative and inflammation by promoting activation of the AKT pathway (Wei et al., 2021). To explore whether BRD4 inhibition mitigates H_2_O_2_-induced damage to AEC-II cells by activating the AKT signaling pathway, an AKT inhibitor, Ly294002, was used to treat **AEC**-II cells before H_2_O_2_ induction. Western blotting results showed that BRD4 inhibition significantly reversed H_2_O_2_-induced inactivation of the AKT signaling pathway, while such an effect was partly abolished by Ly294002 (Figure 3A), indicating BRD4 might inhibit the AKT signaling pathway in H_2_O_2_-challenged **AEC**-II cells.

As shown in Figures 3B,C, Ly294002 pretreatment partly reversed the suppressive effects of BRD4 knockdown on H_2_O_2_-triggered cell viability inhibition and cell damage. Besides, Ly294002 also abolished the ameliorative effects of BRD4 knockdown on H_2_O_2_-induced inflammatory responses (Figures 3D,E) and oxidative stress in **AEC**-II cells (Figures 3F–H). Moreover, the inhibitory effect of BRD4 depletion on H_2_O_2_-induced **AEC**-II cell apoptosis was also abated by Ly294002 treatment (Figures 3I,J). Therefore, these data suggest that BRD4 inhibition ameliorates H_2_O_2_-induced cell **AEC**-II cell damage by activating the AKT signaling pathway.

### BRD4 knockdown reverses SIRT3 inhibition in H_2_O_2_-challenged AEC-II cells via AKT activation

**FIGURE 4:**
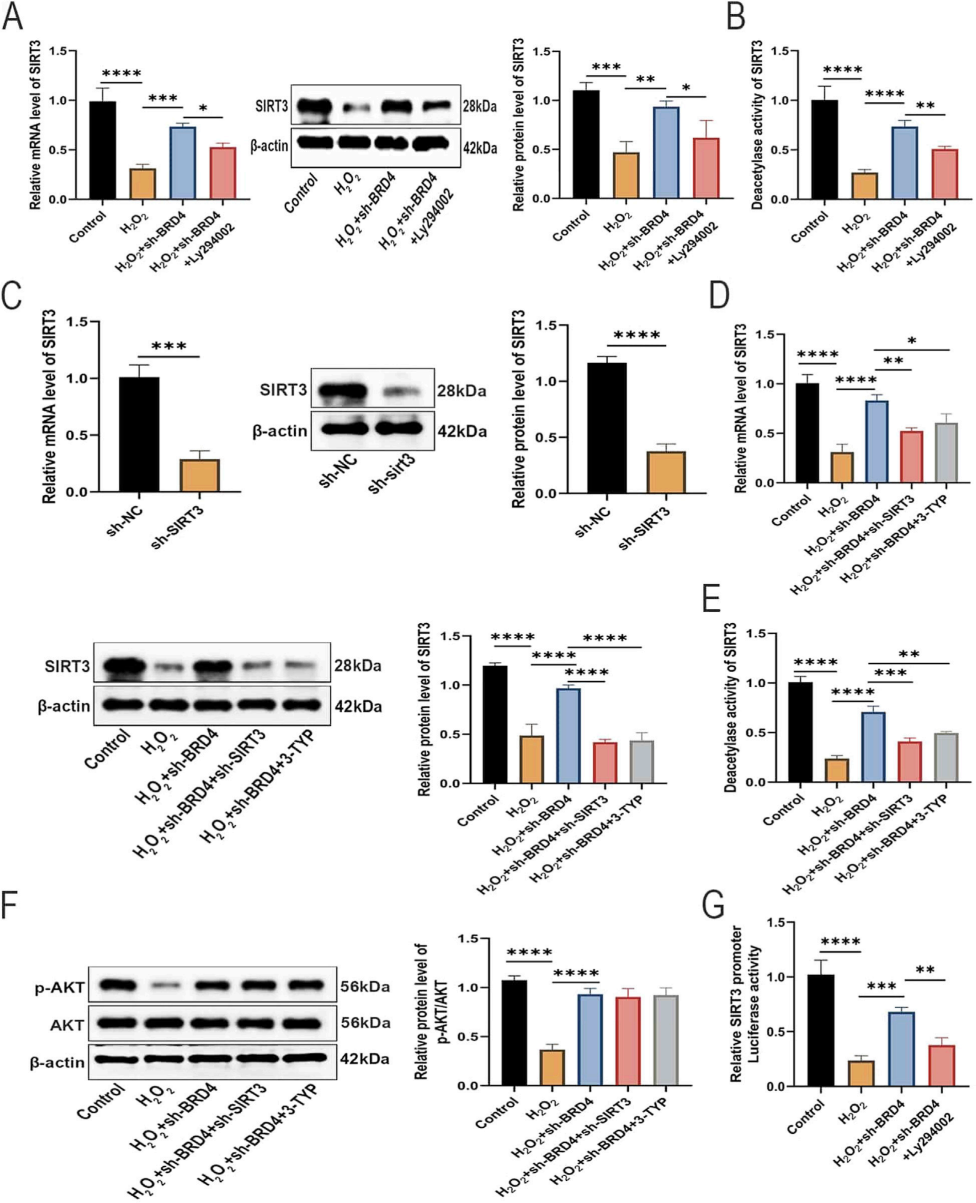
BRD4 knockdown reverses SIRT3 inhibition in H_2_O_2_-challenged AEC-II cells via AKT activation. **(A)** SIRT3 mRNA and protein expressions in AEC-II cells from Control, H_2_O_2_, H_2_O_2_+sh-BRD4, and H_2_O_2_+sh-BRD4+Ly294002 group. **(B)** SIRT3 deacetylase activity in AEC-II cells from Control, H_2_O_2_, H_2_O_2_+sh-BRD4, and H_2_O_2_+sh-BRD4+Ly294002 group. **(C)** SIRT3 mRNA and protein expressions in AEC-II cells transfected with sh-SIRT3 or sh-NC. Then, AEC-II cells were assigned to Control, H_2_O_2_, H_2_O_2_+sh-BRD4, H_2_O_2_+sh-BRD4+sh-SIRT3, and H_2_O_2_+sh-BRD4+3-TYP groups. **(D)** SIRT3 mRNA and protein expressions in AEC-II cells from each group. **(E)** SIRT3 deacetylase activity in AEC-II cells from each group. **(F)** The p-AKT and AKT protein levels in AEC-II cells from each group. **(G)** SIRT3 promoter activity in each group was assessed by dual-luciferase reporter assay. Cell experiments were performed in triplicates (n = 3). *P < 0.05; **P < 0.01; ***P < 0.001; ****P < 0.0001.

As reported in previous studies, SIRT3 is lowly expressed and plays a protective role in HILI (Tian and Zhang, 2018). In addition, AKT, an upstream regulator of SIRT3, is required for SIRT3 stabilization (Liu et al., 2022). Therefore, it was speculated that SIRT3 may be also involved in the protection of BRD4 inhibition against HILI. As expected, BRD4 knockdown significantly reversed H_2_O_2_-triggered inhibition on SIRT3 expression and SIRT3 deacetylase activity in **AEC**-II cells, while Ly294002 partially abolished such an effect (Figures 4A,B). As illustrated in Figure 4C, Western blotting and RT-qPCR confirmed the SIRT3 knockdown efficiency in **AEC**-II cells. Western blotting results revealed that SIRT3 knockdown or pharmacological inhibition by SIRT3 inhibitor, 3-TYP, significantly abated the promoting effects of BRD4 silencing on SIRT3 expression and SIRT3 deacetylase activity (Figures 4D,E). Interestingly, p-AKT expression was not altered in response to SIRT3 inhibition, indicating that SIRT3 is a downstream molecule of AKT (Figure 4F). BRD4 has been identified as a negative regulator of SIRT1 expression by inhibiting its transcriptional activity (Liu et al., 2024). Presumably, BRD4 may regulate SIRT3 expression by modulating its promoter activity. As illustrated by the results of dual-luciferase reporter assays, BRD4 knockdown increased SIRT3 promoter activity, which was partly reversed by LY294002 (Figure 4G), indicating that BRD4 epigenetically repressed SIRT3 expression by reducing SIRT3 promoter activity via the upstream AKT pathway. Collectively, BRD4 inhibition upregulated SIRT3 expression in H_2_O_2_-treated **AEC**-II cells by promoting its transcription via AKT activation.

### SIRT3 inhibition reverses the protective effects of BRD4 inhibition on H_2_O_2_-challenged AEC-II cells

**FIGURE 5:**
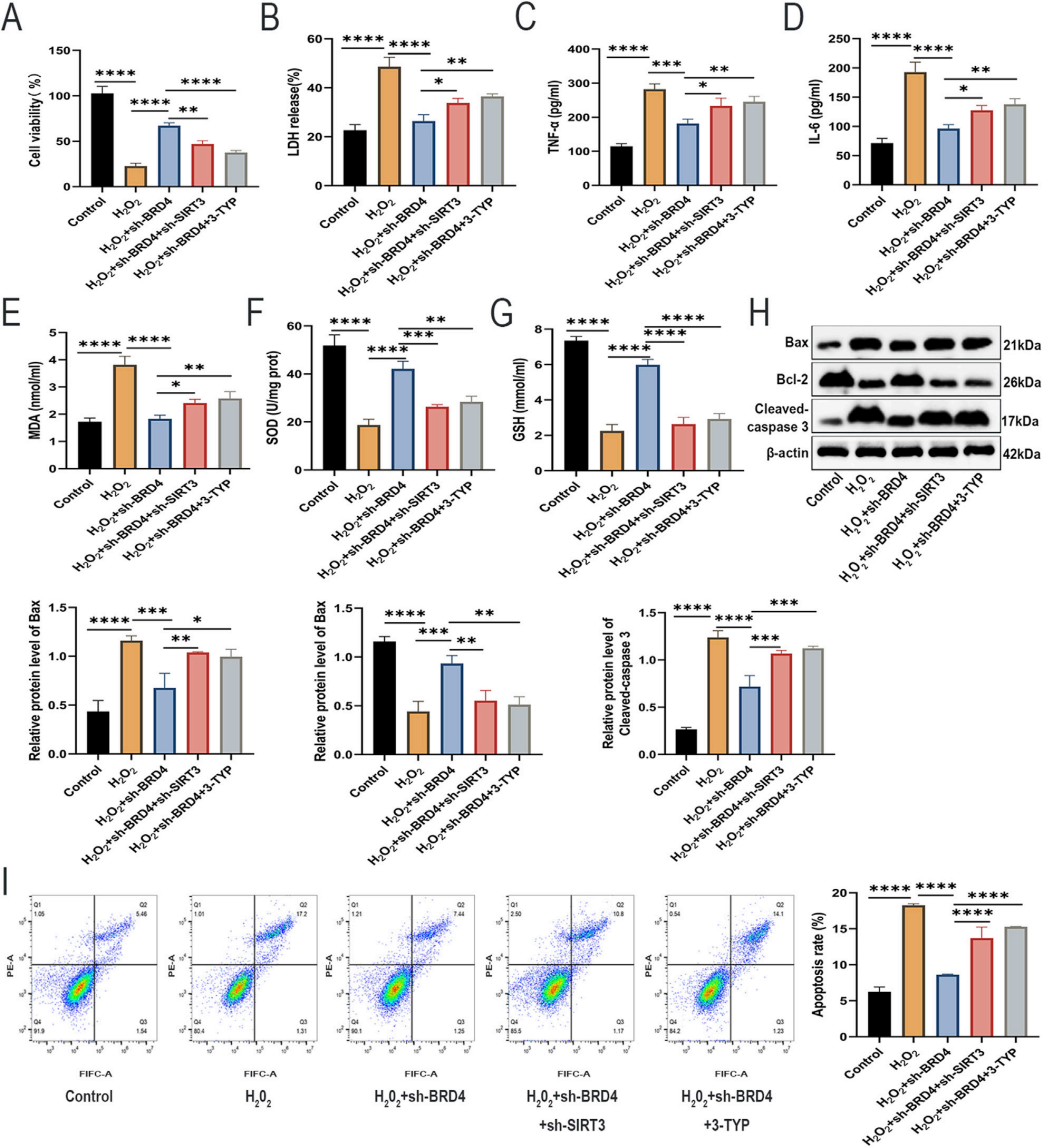
SIRT3 inhibition reverses the protective effects of BRD4 inhibition on H_2_O_2_-challenged AEC-II cells. **(A,B)** The viability and LDH release of AEC-II cells from each group. **(C,D)** TNF-α and IL-6 levels in AEC-II cell supernatant from each group. **(E–G)** MDA, SOD, and GSH levels in AEC-II cells from each group. **(H)** Bax, Bcl-2, and cleaved-caspase 3 protein levels in AEC-II cells from each group. **(I)** The apoptosis of AEC-II cells from each group. Cell experiments were performed in triplicates (n = 3). *P < 0.05; **P < 0.01; ***P < 0.001; ****P < 0.0001.

Next, we investigated whether SIRT3 is also involved in BRD4-dependent apoptosis, inflammatory responses, and oxidative stress of H_2_O_2_-challenged **AEC**-II cells. Rescue assays showed that SIRT3 knockdown or pharmacological inhibition significantly abolished the suppressive effects of BRD4 knockdown on H_2_O_2_-induced **AEC**-II cell damage (Figures 5A,B), inflammatory responses (Figures 5C,D), oxidative stress (Figures 5E–G), and apoptosis (Figures 5H,I). Therefore, BRD4 inhibition upregulated SIRT3 expression to reduce H_2_O_2_-triggered damage to **AEC**-II cells.

### BRD4 inhibition mitigates HILI in mice by activating the AKT/SIRT3 signaling

**FIGURE 6:**
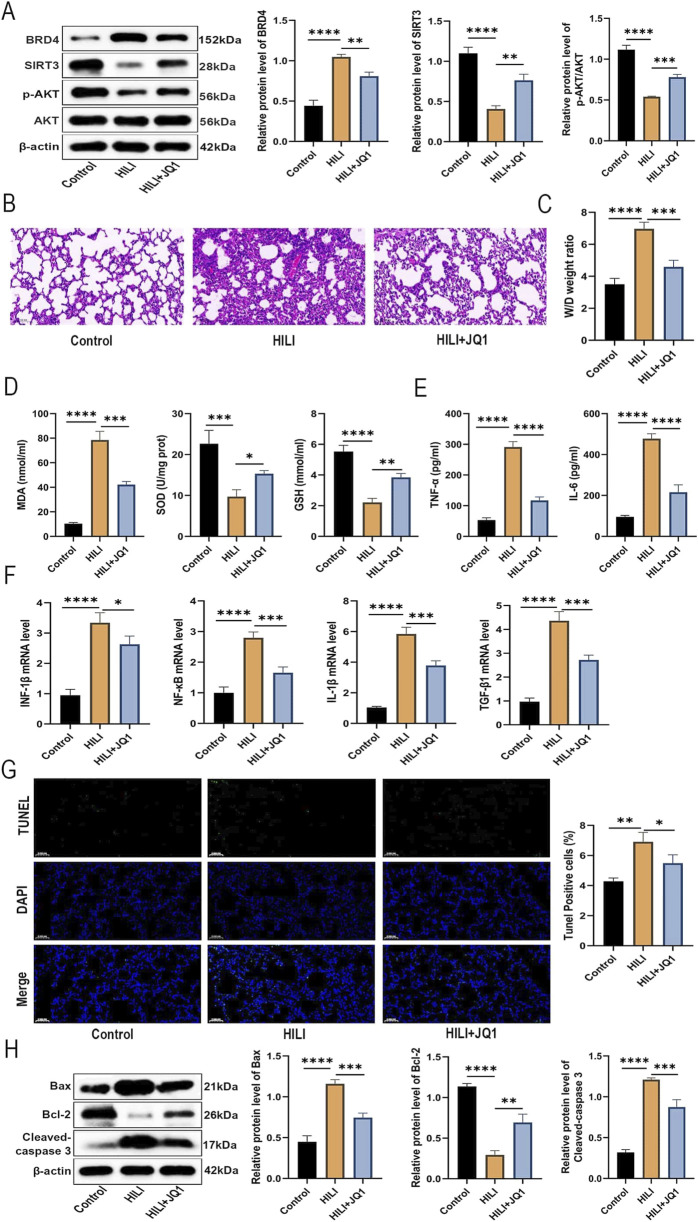
BRD4 inhibition mitigates HILI in mice by activating the AKT/SIRT3 signaling. SD mice were assigned to Control, HILI, and HILI + JQ1 groups. **(A)** BRD4, SIRT3, p-AKT, and AKT protein levels in lung tissues from each group. **(B)** H&E staining of lung tissues from Control and HILI mice (×30.0 magnification; scale bar = 100 µm). **(C)** W/D weight ratio of lung tissues from each group. **(D)** MDA, SOD, and GSH levels in lung tissues from each group. **(E)** TNF-α and IL-6 levels in BALF from each group. **(F)** IFN-β, NF-κB, IL-1β, and TGF-β1 expression in lung tissues from each group was detected by RT-qPCR **(G)** TUNEL analysis of lung sections from each group. **(H)** The relative protein levels of Bax, Bcl-2, and in tissues from each group. *P < 0.05; **P < 0.01; ***P < 0.001; ****P < 0.0001.

To verify the impact of BRD4 inhibition on HILI *in vivo*, a specific BRD4 inhibitor, JQ1, was used. As shown in Figure 6A, JQ1 treatment remarkably abrogated the increase in BRD4 protein level and the reduction of SIRT3 and p-AKT protein levels in lung tissues from HILI mice. According to H&E staining, pharmacological inhibition of BRD4 by JQ1 significantly ameliorated destruction of alveolar structure, different sizes, and thickening of pulmonary septum, relative to the HILI group (Figure 6B). In addition, JQ1 treatment markedly reduced the elevation in W/D weight ratio of lung tissues from HILI mice (Figure 6C). Furthermore, JQ1 treatment substantially reversed the oxidative damage and inflammatory response in lung tissues from HILI mice (Figures 6D–F). As shown in Figure 6G, the number of TUNEL-positive apoptotic cells in lung tissue from HILI mice was significantly higher compared to the control mice, while this increase in apoptosis was markedly inhibited by treatment with JQ1. Western blot analysis of lung tissue extracts further supported these results, showing that JQ1 treatment notably decreased the protein levels of cleaved-caspase 3 and Bax in the lung tissues of HILI mice (Figure 6H). The above results indicated that BRD4 inhibition might mitigate HILI in mice through the activation of the AKT/SIRT3 axis.

## Discussion

HILI is a severe condition characterized by lung inflammation and injury resulting from exposure to high levels of oxygen (Chu et al., 2022). Increased apoptosis and reduced proliferation of AEC-II cells are key factors contributing to HILI (Guo et al., 2024). Apoptosis is the most extensively studied form of cell death and represents the primary mode of cell death during development and in the maintenance of organismal homeostasis (Singh et al., 2019). Both the intrinsic and extrinsic apoptotic pathways can be directly or indirectly modulated by BRD4 (Hu et al., 2022). BRD4 has emerged as a potential therapeutic target for a range of human diseases (Sun et al., 2024), including cancers (Zheng et al., 2022), gastrointestinal diseases (Ma et al., 2023), and cardiovascular diseases (Lin and Du, 2020). Inhibition of BRD4 reduces Pulmonary Hypertension Resulting from Combined Hypoxia and Pulmonary Inflammation (Chabert et al., 2018), inhibited the growth of non-small cell lung carcinoma via AKT activation (Yuan et al., 2023)^.^ BRD4 is increasingly being recognized for its crucial involvement in modulating various physiological and pathological processes associated with pulmonary diseases (Duan et al., 2023; Wang et al., 2021). In the current study, **AEC**-II cells were challenged by H_2_O_2_ to imitate HILI *in vitro*. In addition, we also established a mice model of HILI *in vivo*. Both *in vitro* and *in vivo* experimental results demonstrated a notable rise in BRD4 expression within HILI. These findings indicate that BRD4 may be involved in the progression of HILI.

Studies have implicated the contribution of BRD4 to H_2_O_2_-induced oxidative damage (Zhang and Xu, 2020; Hussong et al., 2014). In addition, BRD4 inhibition also plays a protective role against cell apoptosis, inflammation, and oxidative stress (Shen and Li, 2021; Bie et al., 2023; Song et al., 2023). Herein, we first evaluated the impact of BRD4 inhibition and overexpression in HILI *in vitro*. Through *in vitro* experiments, we found that H_2_O_2_-caused apoptosis, inflammatory response, and oxidative stress of **AEC**-II cells could be reduced by BRD4 inhibition but reinforced by BRD4 overexpression, suggesting that BRD4 inhibition protects against HILI *in vitro*.

A key discovery in this study is the role of the AKT signaling pathway in mediating the protective effects associated with BRD4 inhibition. The AKT pathway is vital for cell survival and is involved in a variety of cellular processes, including apoptosis, inflammation, autophagy and oxidative stress (Song et al., 2019; Fang et al., 2024; Xia et al., 2025; Peng et al., 2019). In cancer, this pathway is frequently disrupted, which promotes tumor development and progression (Ke et al., 2024)^.^ Previous research has identified the protective role of AKT activation in HILI (Ruan et al., 2020; Soundararajan et al., 2022; Cao et al., 2015). Interestingly, BRD4 inhibition has been reported to promote AKT activation (Liu H. et al., 2019; Yang et al., 2024). Herein, we observed that BRD4 inhibition substantially abolished the inactivation of the AKT signaling pathway in H_2_O_2_-treated **AEC**-II cells, which was partially reversed by the AKT inhibitor, Ly294002. In addition, Ly294002 also abrogated the inhibitory effects of BRD4 knockdown on H_2_O_2_-provoked viability inhibition, inflammatory response, oxidative stress, and apoptosis. These results indicate the importance of the AKT pathway in mitigating H_2_O_2_-induced **AEC**-II cell apoptosis, inflammatory response, and oxidative stress.

Furthermore, we identified SIRT3, a mitochondrial deacetylase crucial for oxidative stress regulation (Ren et al., 2016; Trinh et al., 2024), as a downstream target of the BRD4-AKT axis. It has been reported that SIRT3 is downregulated in HILI and exerts a protective role in this condition (Tian and Zhang, 2018). Herein, SIRT3 expression and activity were markedly suppressed in H_2_O_2_-challenged **AEC**-II cells but were restored upon BRD4 knockdown. Importantly, inhibition of AKT attenuated BRD4 knockdown-induced SIRT3 upregulation, indicating that BRD4 regulates SIRT3 expression via AKT activation. BRD4 has been identified as an epigenetic regulator that plays a critical role in the transcriptional process (Szczepanski et al., 2020). Mechanistically, dual-luciferase assays revealed that BRD4 suppression enhanced SIRT3 promoter activity via AKT activation, further substantiating that BRD4 epigenetically regulates SIRT3 transcription and expression via the upstream AKT pathway. Functional studies confirmed that SIRT3 inhibition reversed the protective effects of BRD4 knockdown, underscoring the significance of the AKT-SIRT3 pathway in mitigating HILI.

To corroborate these *in vitro* findings, we evaluated the therapeutic potential of pharmacological BRD4 inhibition in a mice model of HILI. The BRD4 inhibitor JQ1 demonstrated remarkable efficacy in mitigating HILI in mice, which was evidenced by attenuated histopathological damage, edema, inflammation, and oxidative damage, and apoptosis while restoring SIRT3 and p-AKT expression in lung tissues. These findings offer strong evidence supporting the therapeutic potential of targeting BRD4 in HILI.

Despite these promising findings, our study still has some limitations. First, ultrastructural analysis was not performed, which could have provided a deeper understanding of mitochondrial damage and other subcellular changes associated with HILI. Second, while we focused on the AKT-SIRT3 pathway, BRD4 is known to regulate other molecular pathways that may contribute to HILI, and potential off-target effects of BRD4 inhibition require further exploration. Finally, our study primarily assessed the acute effects of BRD4 inhibition, the long-term implications of sustained BRD4 suppression on lung homeostasis and potential systemic side effects or toxicity remain unknown. This necessitates rigorous monitoring in animal models over extended periods to systematically evaluate the impact of prolonged BRD4 inhibitor administration on lung structure and function, as well as on other critical organs such as the heart, liver, kidneys, and immune system. Concurrently, developing inhibitors with higher selectivity for BRD4 may help circumvent potential adverse effects associated with inhibiting other BET proteins (e.g., BRD2, BRD3).

To sum up, our study unveils BRD4 inhibition as an innovative therapeutic approach for mitigating HILI, offering mechanistic insights into its protective mechanisms via activation of the AKT-SIRT3 signaling. Our findings not only deepen our understanding of HILI pathogenesis but also lay the foundation for the development of targeted therapeutic strategies to address this devastating complication.

## Data Availability

The raw data supporting the conclusions of this article will be made available by the authors, without undue reservation.
